# Mebendazole and a non-steroidal anti-inflammatory combine to reduce tumor initiation in a colon cancer preclinical model

**DOI:** 10.18632/oncotarget.11851

**Published:** 2016-09-06

**Authors:** Tara Williamson, Ren-Yuan Bai, Verena Staedtke, David Huso, Gregory J. Riggins

**Affiliations:** ^1^ Department of Neurosurgery, Johns Hopkins University School of Medicine, Baltimore, MD, USA; ^2^ Department of Neurology, Johns Hopkins University School of Medicine, Baltimore, MD, USA; ^3^ Department of Molecular and Comparative Pathobiology, Johns Hopkins University School of Medicine, Baltimore, MD, USA; ^4^ Sidney Kimmel Comprehensive Cancer Center, Johns Hopkins University School of Medicine, Baltimore, MD, USA

**Keywords:** mebendazole, sulindac, FAP, APC, colon cancer

## Abstract

Inheritance of a gene mutation leads to the initiation of 5 to 10% of most cancers, including colon cancer cases. We developed a chemoprevention strategy using a novel combination of the non-steroidal anti-inflammatory (NSAID) sulindac plus the anthelminthic benzimidazole, mebendazole. This oral drug combination was effective in the *Apc^Min/+^* mouse model of Familial Adenomatous Polyposis (FAP). Treatment with 35 mg/kg daily mebendazole reduced the number of intestinal adenomas by 56% (P = 0.0002), 160 ppm sulindac by 74% (P < 0.0001), and the combination by 90% (P < 0.0001). The combination significantly reduced microadenomas, polyp number and size in both the small intestines and colon when compared to untreated controls or sulindac alone. Mebendazole as a single agent decreased COX2 expression, blood vessel formation, VEGFR2 phosphorylation, and worked synergistically with sulindac to reduce overexpression of MYC, BCL2, and various pro-inflammatory cytokines. Given the low toxicity of mebendazole, these preclinical findings support the consideration of clinical trials for high risk cancer patients using mebendazole either alone or in combination. The findings have implications for populations with moderate and above risk for developing cancer.

## INTRODUCTION

Individuals at high risk for cancer, for example those with a germline cancer causing mutation, have limited options to reduce cancer risk. Hereditary cancers represent a significant fraction of cancers. For colorectal cancer (CRC) about 5 to 10% of cases are Mendelian inherited [1].

Familial Adenomatous Polyposis (FAP) is an autosomal dominant inherited form of colon cancer caused by germline mutations in the *Adenomatous polyposis coli* (*APC*) tumor suppressor gene which is characterized, by hundreds of thousands of polyps in the gastrointestinal tract [2]. In FAP patients, benign polyps develop early in the teen years and one or more will transform to colorectal cancer by age 40, if left untreated [3]. Prophylactic colectomy reduces mortality in FAP patients, but not completely and has an associated morbidity.

An animal model for FAP is the C57BL6 *Apc^Min/+^* mouse, which carries a mutation in the murine *APC* gene. The *Apc^Min/+^* mouse has a similar phenotype to FAP, with multiple intestinal adenomas [4]. It is frequently used for preclinical testing of cancer preventative agents.

The *APC* tumor suppressor is the initiating mutation for most cases of colon cancer progression [5]. It functions to determine intestinal cell fate via negative regulation of the β-Catenin/Wnt pathway [6]. Approximately 1% of CRCs are a result of an inherited *APC* mutation and nearly all sporadic CRCs are initiated by APC or β-catenin mutations [2, 7]. Inactivation of *APC* results in activation of *MYC* and *CCND1* (cyclin D1) expression promoting aberrant crypt foci and subsequent microadenoma formation [6, 8, 9].

A risk factor and initiating mechanism for CRC is chronic inflammation of the colon epithelium. Deregulated Prostaglandin-Endoperoxide Synthase 2 (PTGS2, aka COX2) and increased levels of prostaglandin E2 (PGE2) serve as mediators of inflammation and tumorigenesis [10]. These promote β-catenin/TCF4-mediated transcription and upregulation of the anti-apoptosis protein BCL2 [11].

Inflammatory cell infiltration and increased pro-inflammatory cytokines further drive tumor formation and progression [10]. Intestinal polyp formation in the *Apc^Min/+^* mouse coincides with increased pro-inflammatory cytokines such as TNF, IL1B, IL6, and CCL2 (MCP-1), likely driven by constitutively activated NFKB1 [12].

The VEGF/VEGFR2 pathways are also activated during colon tumor progression [10, 14]. VEGF activation contributes to the inflammatory process and promotes angiogenesis [15]. Complex interconnected pathways such as chronic inflammation and sustained angiogenesis work in concert to drive formation and progression of CRC.

Due to redundancy of the processes driving tumor formation, inhibition of multiple targets could be more effective than single targets. Safe and effective drug combinations (and lifestyle changes) that can better decrease inflammation and possibly angiogenesis and/or apoptosis are a potential means to reduce cancer incidence in high risk individuals.

Nonsteroidal anti-inflammatory drugs (NSAIDs), such as sulindac, represent an effective class of compounds for the prevention of colon cancer [16, 17], and a logical starting point for more effective chemo-preventative drug combinations. Preclinical and clinical studies support sulindac's ability to suppress intestinal tumorigenesis via both COX2 dependent and independent mechanisms [16, 18]. However, use of sulindac for chemoprevention is tempered by a risk of gastrointestinal ulceration and adverse cardiovascular events [17]. Sulindac unfortunately shows no benefit in recurrent colorectal cancer, and resistance to sulindac's benefit has been documented in FAP patients [19]. Lower doses of sulindac, combined with a safe and effective agent with a complementary mode of action, has been suggested for colon cancer chemoprevention in high risk patients [20].

Mebendazole (MBZ) is an FDA approved anthelmintic benzimidazole that has shown preclinical anti-cancer activity in a variety of malignancies including colorectal cancer [21, 22], likely through a combination of molecular mechanisms that include tubulin disruption and VEGFR2-mediated anti-angiogenesis [23, 24]. Preclinical investigations have suggested mebendazole might be useful for treatment of colon, brain, lung, and other cancers [21, 23–28]. Case reports of mebendazole repurposing for colorectal and other cancers have been reported and clinical trials repurposing mebendazole for cancer therapy are currently underway [22, 29].

Given mebendazole's safety and lack of toxicity in adult and pediatric patients, multiple anticancer mechanisms and possible effectiveness as a cancer therapy, we hypothesize that mebendazole could be repurposed as a chemoprevention drug for patients at high risk for developing cancer. Since the *Apc^Min/+^* mouse has the most reported data as a colorectal cancer chemoprevention model [30], we chose this model to test mebendazole's ability for chemoprevention. In this study, we confirmed that mebendazole can slow colon cancer xenograft growth when used therapeutically. More importantly, we reveal in this study that mebendazole has the ability to reduce tumor initiation and this effect is most potent in combination with sulindac to inhibit polyp formation in the intestines of the *Apc^Min/+^* mouse via mechanisms that include inhibition of angiogenesis and inflammation.

## RESULTS

### MBZ suppresses tumor growth in CRC cell lines and xenografts

**Figure 1:**
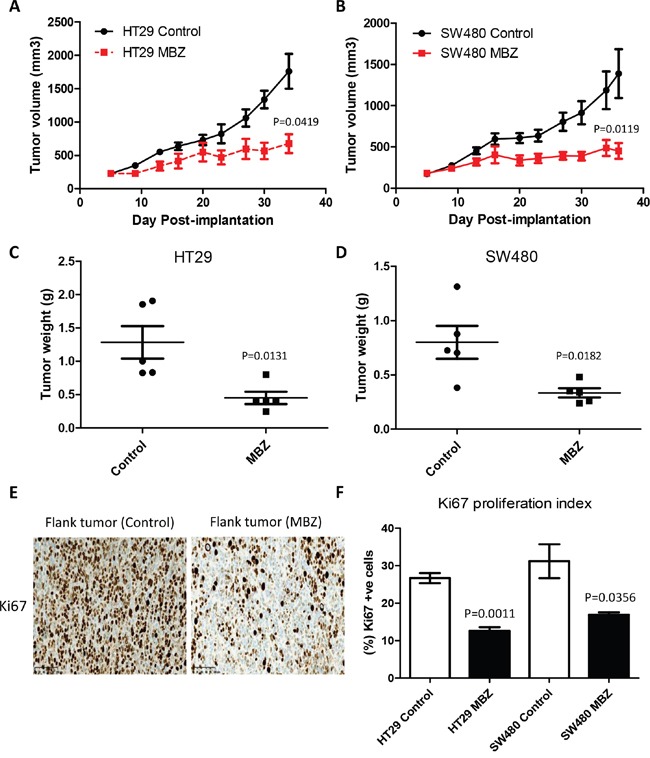
Oral Mebendazole inhibits growth and proliferation in two different colon cancer flank xenografts **A** and **B.** HT29 and SW480 human colorectal carcinoma cell lines, were implanted into the flanks of Nude mice (n=5 per group) and showed a significantly slower growth rate over four weeks of treatment with 50 mg/kg oral MBZ compared to untreated controls. **C** and **D.** Individual resected flank tumors from each group were weighed at the end of the experiment and compared to untreated control showing a decreased final weight after MBZ treatment. **E.** Paraffin-embedded flank tumor sections were stained for Ki67 proliferation marker. Five randomly selected fields from each slide (n=3 tumors per treatment group) were quantified as the percent Ki67-positive cell x 100/total number of cells and represent the mean + SEM. HT29 flank tissue is shown. **F.** MBZ treated tissue showed significantly less positive (brown nuclei) staining in both models.

The MBZ half maximal inhibitory concentrations (IC_50_) for DLD-1, HCT-116, HT29 and SW480 were 0.28 μM, 0.25 μM, 0.20 μM, and 0.81 μM respectively, similar to previous data [21]. Flank xenografts of HT29 and SW480 were used to assess MBZ efficacy (Figure 1A-1D). In the HT29 flanks, the MBZ treated tumors were 62% smaller by volume (1759 mm^3^ vs. 675 mm^3^, P = 0.0419) and 65% smaller by weight (1.28 g vs. 0.45 g, P=0.0131) compared to control. In the SW480 flanks, the MBZ treated tumors were 67% smaller by volume (1389 mm^3^ vs 452 mm^3^, P = 0.0119) and 59% smaller by weight (0.80 g vs. 0.33 g, P = 0.0182) compared to control. Paraffin-embedded flank tissue sections (n=3 tumors per treatment group) were stained with Ki67 and the average percentage of positively stained nuclei were calculated from five randomly selected fields (20X) per tumor. Expression of Ki67 was significantly lower in the MBZ treated tissue versus untreated tissue in both xenografts (HT29 P = 0.0011, SW480 P = 0.0356) (Figure 1E and 1F). This work and previously published data strongly indicates that MBZ inhibits colon cancer cells [21]. We next investigated a more novel hypothesis, if MBZ would work as a chemopreventative.

### MBZ treatment reduces intestinal tumorigenesis in the *Apc^Min/+^* mouse

**Figure 2:**
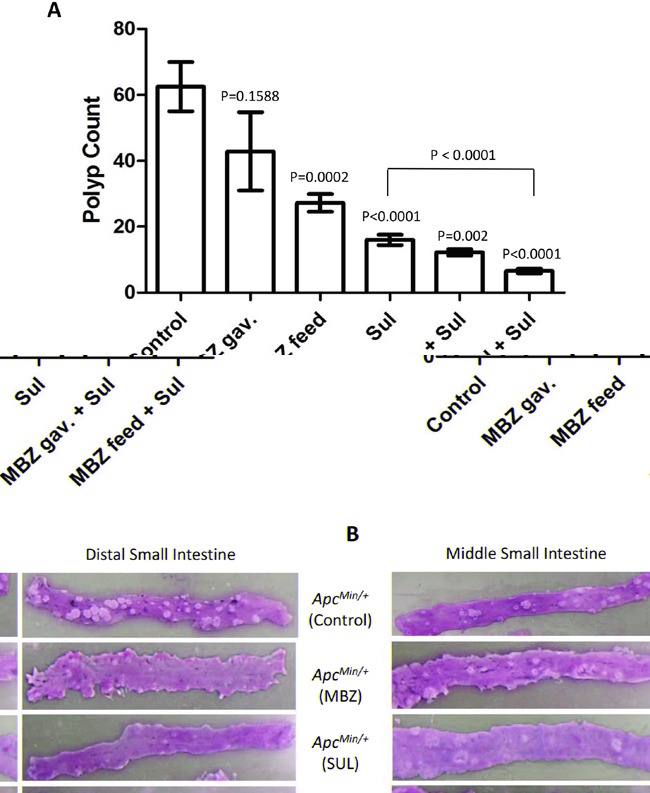
Mebendazole reduces the formation of polyps in the intestine of *Apc^Min/+^* mice **A.** All mouse pups harboring the *Apc^Min/+^* mutation were weaned onto a high fat diet at 3 weeks of age and randomized into treatment groups. Sulindac was provided in buffered drinking water (0.16g/L) starting at 3 weeks of age. MBZ treatment was initiated at 5 weeks of age and was provided in a high-fat custom feed at 35 mg/kg seven days a week or administered by oral gavage at 50 mg/kg five days per week. At 100 days of age, the small intestines and colon of *Apc^Min/+^* mice were analyzed and the total number of polyps/mouse were averaged and compared across treatment groups. **B.** Representative tissue from middle and distal small intestines are shown to compare polyp burden in untreated control, 35 mg/kg MBZ in feed, Sulindac 160 ppm and 35 mg/kg MBZ in feed + 160 ppm Sulindac treatment groups.

Oral mebendazole prevented tumor formation in the intestine of *Apc^Min/+^* mice and the best chemoprevention occurred when MBZ was combined with sulindac (Figure 2A). Compared with the total number of tumors in the intestine of untreated *Apc^Min/+^* mice (62.5 ± 7.50), 35 mg/kg MBZ continuous dose administered in the feed for 9 weeks significantly reduced the number of tumors by 56% (27.25 ± 2.68, P = 0.0002) as a single agent. We also administered MBZ at 50 mg/kg five days per week for 9 weeks via oral gavage to mimic the dosing regimen successfully used in brain tumor chemotherapy [23], but found that this treatment schedule did not produce a significant outcome (P = 0.1588) and there was a high degree of variability in the final tumor counts (42.86 ± 11.88). Sulindac alone at a dose of 160 ppm in the drinking water for 11 weeks inhibited polyp formation by 74% (16 ± 1.57, P < 0.0001) as expected based on previous reports [18, 30, 31]. However, the combination of sulindac plus MBZ produced an even greater chemoprotective effect, reducing polyp number by 80% (12.25 ± 0.95, P=0.002) when 50 mg/kg MBZ gavage was used and by 90% (6.58 ± 0.73, P < 0.0001) when 35 mg/kg MBZ in feed was used. These results indicate that MBZ is chemoprotective as a single agent and works synergistically with sulindac. The addition of MBZ to sulindac reduced tumor formation compared to the current standard of care of sulindac alone (P<0.0001). A summary of total polyp counts and distribution for the entire study can be found in Table 1. Macrodissection of intact intestinal tissue allowed us to quantify polyp number and size and compare tumor burden between treatment groups, with representative pictures shown in Figure 2B.

**Table 1:** Number, size and distribution of polyps for different treatments vs. control

	Region of Intestine (Polyp count ± S.E.M.)						Number of Polyps in size (mm) range			
*Apc^Min/+^* mouse groups	Proximal	Middle	Distal	Colon	Total (% inhibition)	P valueǂ	< 1.0	1.0-2.0	2.1-3.0	> 3.0
Untreated Control	15.5 ± 2.2	28.8 ± 3.7	16.9 ± 2.3	1.25 ± 0.33	62.5		32.8	19.8	7.3	2.7
MBZ 50 mg/kg gavage^*^	9.57 ± 2.41	18.7 ± 6.1	13.9 ± 3.9	0.71 ± 0.29	42.9 (31%)	0.16	24.4	14.6	3.7	0.14
MBZ 35 mg/kg feed	4.58 ± 0.65	12.4 + 1.2	9.83 ± 1.4	0.42 ± 0.19	27.3 (56%)	0.0002	10.6	12.0	4.5	0.45
SUL 160 ppm drinking water	4.77 ± 0.68	6.08 ± 0.94	3.62 ± 0.53	1.54 ± 0.35	16.0 (74%)	<0.0001	8.5	6.1	1.2	0.15
MBZ 50 mg/kg + SUL 160 ppm	3.00 ± 1.35	3.00 ± 0.0	4.25 ± 0.63	2.00 ± 0.41	12.3 (80%)	0.002	7.6	2.8	0.8	0
MBZ 35 mg/kg + SUL 160 ppm	2.42 ± 0.41	2.42 ± 0.37	1.37 ± 0.30	0.37 ± 0.14	6.58 (90%)	<0.0001	3.7	2.6	0.2	0.05

* Gavage at 50 mg/kg was for 5 days per week, with no drug on weekends.

ǂ All p values compared to control.

### MBZ and MBZ plus sulindac inhibit tumor formation in each intestinal segment

**Figure 3:**
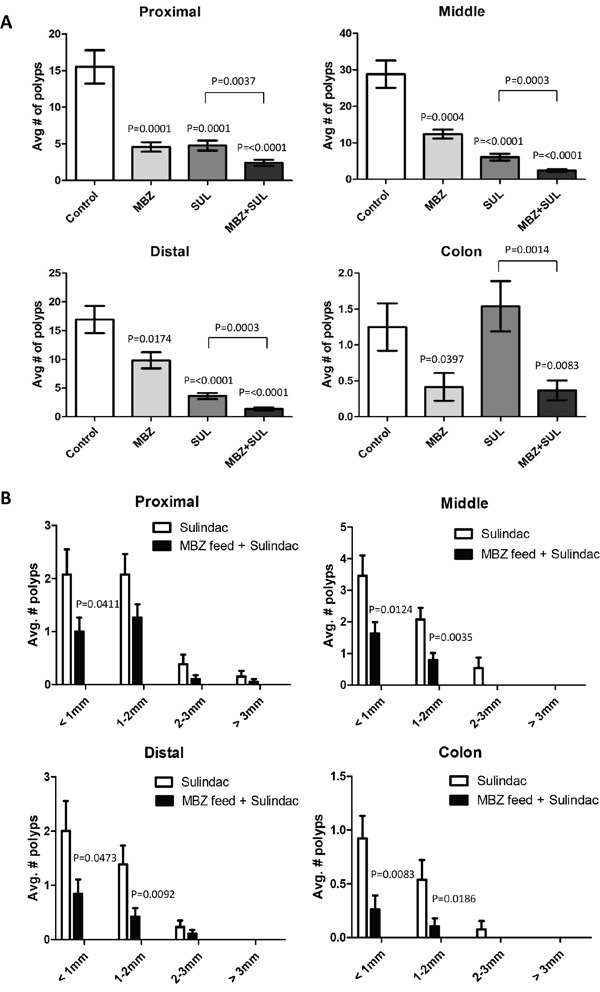
The combination of low dose MBZ plus sulindac act synergistically in reducing both the occurrence and size of tumors in all segments of the *Apc^Min/+^* mouse intestine **A.** The average number of polyps for each treatment group were graphed for the proximal, middle and distal small intestines and colon. P values shown are for treated vs control. **B.** Individual polyps were measured and categorized based on size. The average number of polyps for sulindac versus the combination of MBZ + sulindac were analyzed separately for each section of the intestine. P values indicate significance in MBZ+SUL vs SUL.

We observed that MBZ is most effective in the *Apc^Min/+^* model when administered at 35 mg/kg daily continuous oral dose in the feed, a reasonable dosing regimen for long term chemoprevention. The average distribution of polyps in the small intestine of the untreated control *Apc^Min/+^* mice (Figure 3A) was: proximal (15.50 ± 2.28), middle (28.83 ± 3.74), distal (16.92 ± 2.37). In the proximal small intestine, there was a 70% (4.58 ± 0.65, P = 0.0001) reduction in polyps with MBZ and a 69% (4.77 ± 0.68, P=0.0001) reduction using sulindac compared to control. In the middle segment of the small intestine, there was a 57% (12.42 ± 1.22, P=0.0004) reduction in polyps with MBZ and a 79% (6.08 ± 0.94, P<0.0001) reduction with sulindac compared to control. In the distal small intestine, polyps were reduced by 42% (9.83 ± 1.40, P=0.0174) with MBZ and by 79% (3.62 ± 0.53, P<0.0001) with sulindac compared to control. The combination of sulindac + MBZ had the most potent inhibitory effect in the small intestine by reducing polyp formation by 84% in proximal (2.42 ± 0.41, P<0.0001), 92% in middle (2.42 ± 0.37, P<0.0001), 92% in distal (1.37 ± 0.30, P<0.0001) compared to control. The combination of the two drugs significantly outperformed single agent therapy in every segment.

MBZ alone was also compared to SUL alone (not shown), with SUL overall being more effective than MBZ (p = 0.0012). However this was due to the relative efficacy of SUL in the small intestines and the larger number of polys in the small versus the large intestines in this model. MBZ alone was more effective in the colon compared to SUL (p = 0.0118).

### MBZ mitigates the tumorigenic effect of sulindac in the *Apc^Min/+^* mouse colon

MBZ treatment is more effective in preventing polyp formation in the colon of the *Apc^Min/+^* mouse compared to sulindac. In our study, mice who received sulindac alone experienced a 23% increase in average polyp incidence compared to untreated mice (sulindac = 1.54 vs Control = 1.25). A similar increase in colon polyps due to sulindac has been observed by multiple researchers using the *Apc^Min/+^* mouse [32]. Our results show that MBZ alone decreased tumor burden by 66% (0.42 ± 0.19, P=0.0397) and mitigated tumorigenic effects of sulindac leading to a decrease of 70% (0.37 ± 0.14, P=0.0083) in the MBZ + sulindac combination treatment group, as seen in Figure 3A. The colon is the primary site of polyp formation in human FAP patients, and it would of course be favorable to have a therapy that is more effective in the colon.

### Sulindac plus MBZ eliminates larger polyps and microadenomas

**Figure 4:**
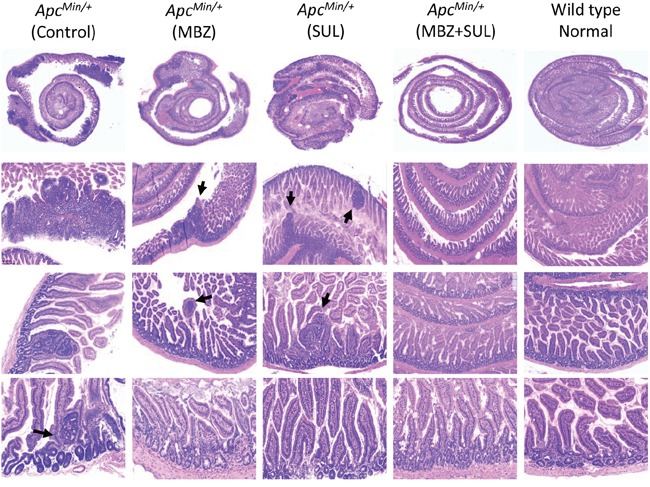
The combination of MBZ plus sulindac reduces the formation of microadenomas Swiss-rolled small intestines were H&E stained and pictures were captured at 1X, 5X, 10X, and 20X to compare the histology between untreated control, MBZ, sulindac, and MBZ + sulindac, and *APC* wild type C57BL6 age-matched littermate (normal control). Histopathological analysis at high magnification shows a decrease in the presence of adenoma and microadenoma (arrows) formation in the MBZ + sulindac combination group.

Table 1 summarizes the average number of polyps in each treatment group that fall within a particular size range. 35 mg/kg MBZ reduced the number of larger polyps compared to control. As with polyp multiplicity, we saw a combination effect between the two drugs to significantly suppress the formation of visible adenomas. The four panels in Figure 3B allow an up close comparison of sulindac as a single agent versus MBZ + sulindac in each segment of the intestine. Combination treatment resulted in significant reduction in the smallest polyps (<1 mm) in all intestinal segments and significant reduction in the 1-2 mm polyps in the middle, distal, and colon when compared to sulindac treatment alone. MBZ + sulindac combination therapy resulted in a total elimination of all polyps over 2 mm in the middle small intestine and colon. H&E stained intestinal segments from each treatment group were analyzed for microadenoma formation by an independent, board-certified veterinary pathologist (D.Huso). The conclusion was that sulindac and MBZ both reduced microadenoma formation versus control but that the MBZ + sulindac combination treatment was most effective with no microadenomas by histology of the sections examined (Figure 4).

### MBZ treatment suppresses oncogenes that drive proliferation and survival

**Figure 5:**
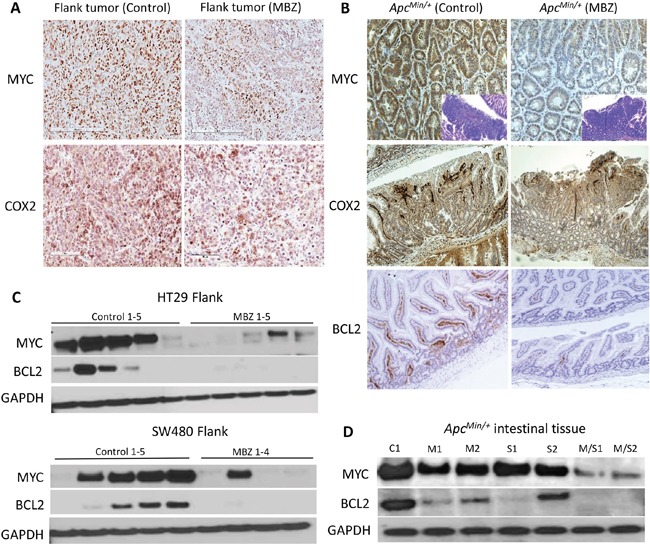
Mebendazole reduces MYC, COX2 and BCL2 in treated *Apc^Min/+^* mouse polyps and in flank xenografts **A.** Paraffin-embedded sections of flank tumor tissue were analyzed by immunohistochemistry using MYC and COX2. **B.** *Apc^Min/+^* polyps were stained for MYC, COX2, and BCL2 showing a reduction for each when mice were fed MBZ, compared to control. **C.** Lysates from individual HT29 (control n=5, MBZ n=5) and SW480 (control n=5, MBZ n=4) flank xenograft tissue were analyzed for MYC and BCL2 protein expression revealing a reduction of these proteins in most cases with MBZ treatment. **D.** Similarly, in the intestines of the *Apc^Min/+^* mouse, there was a reduction of MYC, and BCL2 especially with the combination treatment. C1=control; M1, M2 = MBZ treated; S1, S2 = sulindac treated; M/S1, M/S2 = MBZ + sulindac combination treatment. GAPDH was used as the loading control.

Immunohistochemistry of HT29 flank xenograft tissue and *Apc^Min/+^* intestinal tissue with MYC, COX2, and BCL2 antibodies showed that MBZ treatment had an antitumor effect. Comparing untreated tissue versus 50 mg/kg/day MBZ for four weeks treated tissue showed a 71% reduction in MYC and a 32% reduction in COX2 expression (Figure 5A). *Apc^Min/+^* intestinal polyps that received MBZ treatment at 35 mg/kg daily showed a 35% decrease in MYC expression and a 19% reduction in COX2 expression. BCL2 expression in the intestinal villus was reduced 88% after MBZ treatment (Figure 5B). Lysates from flank and intestinal tissue were probed using MYC and BCL2 antibodies via western blotting. The intensity of the bands for each group were averaged and values were expressed as percent change for control versus treated. In HT29, there was an 83% decrease in MYC and no detectable BCL2 expression after MBZ treatment. In SW480, there was a 75% decrease in MYC, and again, no detectable BCL2 after MBZ treatment (Figure 5C). We then looked at expression of these proteins in control versus treated *Apc^Min/+^* intestinal tissue lysate. For MYC, there was a 29% decrease with MBZ, a 22% decrease using sulindac, and a 78% decrease using MBZ + sulindac. For BCL2, there was a 69% decrease with MBZ alone, a 73% decrease using sulindac alone, and a 98% decrease using the MBZ + sulindac combination treatment (Figure 5D). *Apc^Min/+^* mice with a large polyp burden develop splenomegaly so, as a result, spleen weight is a useful surrogate marker of polyp load [33]. In our experiment, the average spleen size for the Control group, MBZ feed, sulindac, MBZ feed + sulindac and wild-type age matched mice were 406, 207, 126, 108, and 70 mg, respectively. Reduction in MYC has been shown to reverse splenomegaly in *Apc^Min/+^* mice and protect from tumorigenesis, as seen in the mutant *Apc^Min/+^*MYC*^+/−^* mouse model [34]. The present data demonstrate that MBZ is effective as a single agent and in combination with sulindac at reducing expression of proteins that are critical for early intestinal adenoma initiation and tumor progression.

### MBZ impairs tumor angiogenesis and inhibits VEGFR2 kinase activity

**Figure 6:**
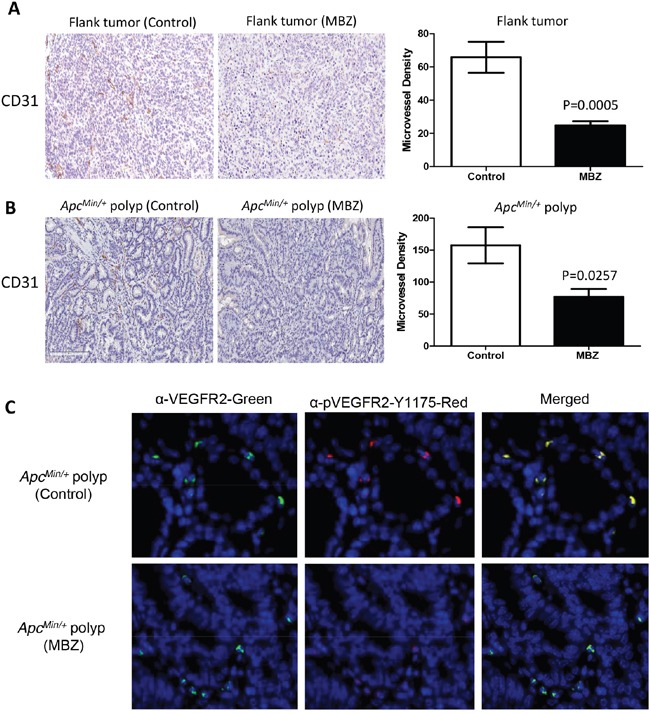
MBZ inhibits VEGFR2 kinase activity and reduces tumor blood vessel formation Paraffin-embedded flank and *Apc^Min/+^* polyps were stained using α-CD31 primary antibody to allow quantification of microvessel density (MVD). **A.** For flank tumors, microvessels were counted and averaged for 10 hotspot fields at 20X. **B.** Polyps in untreated control and MBZ treated *Apc^Min/+^* mice (n=6 polyps per group, average size 1.38 mm) were stained for CD31 and hotspots were counted at 20X **C.** Immunofluorescent staining in control and MBZ treated *Apc^Min/+^* polyps shows mild inhibition of VEGF receptor 2 kinase activity. Anti-VEGFR2 (left panel, green) and anti-p-VEGFR2-Y1175 (middle panel, red), and merged pictures to indicate co-staining (right panel, yellow). All pictures were taken with 800 msec exposures to green, 1500 msec exposure to Texas Red, and 200 msec to DAPI channel.

VEGF signaling is a critical pathway in angiogenesis; it is upregulated in tumors, controls endothelial cell proliferation, neovascular survival, and vascular permeability by binding to the VEGF-receptor 2 (VEGFR2) [33]. MBZ has been previously shown to reduce CD31-positive microvessel formation in non-small cell lung cancer xenografts and in orthotopically implanted medulloblastomas [24, 35]. MBZ inhibits the tyrosine kinase function of VEGFR2 by blocking autophosphorylation at the Y1175 binding site [24]. We hypothesized that inhibiting neovascularization might be a mechanism for MBZ to suppress polyp formation or growth. Anti-CD31 (PECAM-1) endothelial marker was used to assess microvessel formation in flank and intestinal *Apc^Min/+^* tumors. There was a 63% inhibition (P=0.0005) in microvessel density in flank tumors and 51% inhibition (P=0.0257) of microvessel density in MBZ treated *Apc^Min/+^* polyps versus untreated controls (Figure 6A and 6B). *Apc^Min/+^* tumors were dual-stained for VEGFR2 and pVEGFR2-Y1175 and immunofluorescent secondary antibodies were used to visualize a mild reduction in VEGFR2 auto-phosphorylation (Figure 6C).

### Sulindac plus MBZ inhibits inflammatory cytokines and angiogenesis signals in *Apc^Min/+^* mice

Chronic inflammation within the tumor microenvironment can drive tumor progression and promote accumulation of additional mutations and epigenetic changes [10]. An increase in inflammatory stress has been reported to correlate with the development of intestinal polyposis in *Apc^Min/+^* mice. Starting at 12 weeks of age, the polyp burden dramatically increases along with levels of cytokines IL6, TNF, IL1B, and CCL2 [12]. Once the tumor vasculature has been established, the stroma is infiltrated by macrophages that drive cytokine production. Since MBZ has the ability to prevent tumor vessel formation, we hypothesized that it may decrease inflammatory cytokines that can accumulate in the *Apc^Min/+^* intestine.

**Figure 7:**
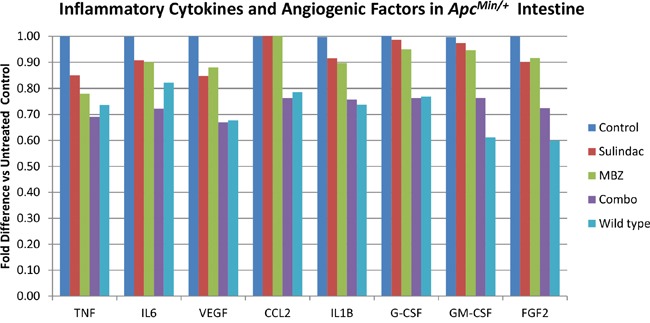
The combination of MBZ plus sulindac decrease inflammatory cytokines and angiogenic factors in the *Apc^Min/+^* intestine more than either drug alone A colorimetric Mouse ELISA strip reactive to TNF, IL6, VEGF, CCL2, IL1B, G-CSF, GM-CSF, and FGF2 was used to measure the reduction of pro-inflammatory markers in each treatment group. The relative absorbance values were averaged and the percent difference in values was compared to the results of the untreated control mice (n=3 mice averaged for each treatment group).

To examine the effects of MBZ alone and MBZ + sulindac combination on various pro-inflammatory cytokines and pro-angiogenic factors, we used a colorimetric Mouse ELISA strip reactive to TNF, IL6, VEGF, CCL2, IL1B, G-CSF, GM-CSF, and FGF2 (Figure 7). The relative absorbance values were averaged for each treatment group (n=3 mice per group) and the percent difference in values was compared to the results of the untreated control mice. In the small intestine, MBZ alone decreased the levels of TNF (22%), IL6 (10%), VEGF (12%), IL1B (10%), G-CSF (5%), GM-CSF (5%), FGF2 (8%). MBZ + Sulindac decreased the levels of TNF (31%), IL6 (28%), VEGF (33%), CCL2 (24%), IL1B (24%), G-CSF (24%), GM-CSF (24%), and FGF2 (28%). In most cases, the combination treatment reduced the cytokines to values that were very similar to the levels observed in wild type littermate controls.

## DISCUSSION

We observed that treating *Apc^Min/+^* mice with the mebendazole plus sulindac combination resulted in 90% less polyps versus untreated mice. At the microscopic level, there was close to normal histopathology and no microadenomas could be found in the combination treated *Apc^Min/+^* mice examined, suggesting that this drug combination acts by preventing tumor initiation, accounting for the 90% reduction. The simplest explanation to account for the 10% adenomas remaining, is that they are the fraction that initiated prior to the start of therapy at 5 weeks.

The size of the remaining treated adenomas was also reduced, consistent with mebendazole's ability to slow tumor growth. There was a ∼50-fold reduction in the largest (3 mm) adenomas (Table 1). In each segment of the intestines there was a reduction in the number and size of tumors for the combination therapy, compared to either control or sulindac alone. Our findings were consistent with previous publications showing that sulindac alone is not effective in preventing colon adenoma formation in the *Apc^Min/+^* mouse and may promote tumorigenesis in the colon, despite being effective in the small intestines [32]. When comparing MBZ alone to the combination, MBZ alone was as effective in the colon, and only in the small intestines was the addition of sulindac helpful. In the colon, the MBZ + sulindac combination treatment group had a four-fold reduction of adenomas relative to sulindac alone and a three-fold relative reduction compared to untreated control mice.

What are possible mechanisms for this combination of a NSAID and mebendazole that allow a significant reduction of adenoma initiation and growth? Evidence here supports a reduction of both inflammation and neovascularization. Sulindac and other NSAIDs work to prevent colon cancer through an inhibition of COX2 [17, 31]. We observed that mebendazole alone also reduces COX2 in the *Apc^Min/+^* mouse adenomas, and even more so when combined with sulindac. Importantly, the combination reduces inflammatory cytokines in the intestinal epithelium more than either treatment alone, to levels very similar to those in *APC* wild type mice. Although there were scant adenomas left for analysis from the combination treatment, the reduction of COX2, MYC, and BCL2 in the mebendazole only tumors also points to reduced inflammation beyond sulindac alone.

Why should mebendazole, whose best documented mechanism is tubulin inhibition [36, 37], inhibit COX2 or inflammation? One possibility is revealed in Figure 6, where mebendazole inhibits VEGFR2 phosphorylation and reduces tumor vascularization (as shown by CD31 staining) in adenomas. We have shown previously in medulloblastoma xenografts that MBZ works via angiogenesis inhibition [24]. The molecular target for mebendazole's kinase inhibition is the ATP binding site on VEGFR2, thus inhibiting its activation [24]. This off-target effect is in addition to its tubulin binding. Whereas it might be difficult to explain how a tubulin inhibitor can prevent adenoma formation, inhibition of VEGF and anti-angiogenesis has a more readily understood mechanism. In addition to lack of vessel growth starving the nascent tumors of oxygen and nutrients, VEGF pathway inhibition itself has an anti-inflammatory effect [15], which likely acts at an earlier stage of tumorigenesis. This model of combined inhibition of inflammation and angiogenesis fits the existing data and is supported by previous mechanistic studies [24].

The important considerations for a chemo-preventative drug are safety, efficacy and lack of toxicity. Mebendazole is relatively non-toxic with a 44-year track record of safe use, with millions of patients who have taken the drug. In many regions of the world mebendazole is sold over the counter. We achieved the best results with continuous dosing of mebendazole in the feed, with an effective dose averaging 35 mg/kg/day. The 5 day a week oral gavage, did not work as well despite an equivalent weekly dose, perhaps due to the interruption in therapy. Daily oral dosing of 35 mg/kg or more is readily achievable in humans where up to 200 mg/kg/day have been used for years of hydatid disease therapy [38] and have been used during a phase 1 trial for newly diagnosed glioblastoma. We recommend a dose range of 40 to 75 mg/kg/day of mebendazole in humans for chemoprevention, along with monthly monitoring of blood counts and serum chemistry including liver function enzymes.

The toxicity of NSAIDs for use in this therapy is of far greater concern. Sulindac was chosen as a positive control because it had the best supporting data showing efficacy in the *Apc^Min/+^* mouse, has been demonstrated in humans, and it is as close to a standard of care for FAP patients as possible [39]. However, unlike mebendazole, its potential cardiac side effects or bowl perforations can be lethal. We noted in our study that the combination of MBZ + sulindac reduced sulindac's toxicity, with no weight loss and other side effects compared to sulindac alone. Despite this positive effect of mebendazole to both increase efficacy and reduce apparent toxicity of sulindac, it will be prudent to investigate other NSAID/mebendazole combinations or even consider the use of mebendazole alone for colon adenoma prevention given it's favorable efficacy to toxicity ratio.

We do not think other anti-parasitic benzimidazoles will work as well as mebendazole, based on our empirical preclinical testing of most of the approved drugs in this class. However, in this study we did employ an improved mebendazole formulation of pure polymorph C [40]. Although we have not directly compared different formulations of mebendazole in the *Apc^Min/+^* mouse, the polymorph C version of mebendazole is better absorbed than the other polymorphs or several generic versions of mebendazole, yielding more favorable pharmacokinetics and better anti-tumor efficacy [40].

All versions of mebendazole can be obtained at low cost, and over the counter in many countries. This is an advantage to reach larger numbers of patients, in particular in economically underprivileged populations.

While our testing and molecular investigation of the combination of mebendazole plus NSAID has been limited to the *Apc^Min/+^* mouse model of FAP, the implications of these results are broader. Many colon and other cancer types are initiated or driven at least in part by inflammation. Colon cancer is common and there are other high risk populations where the benefits of this therapy would outweigh risk.

While further mechanistic and translational development of employing mebendazole plus NSAID for chemoprevention are warranted, it is not too early to consider pilot clinical studies for the FAP patient. These patients are in immediate need of more effective chemoprevention strategies. A clinical study of the effect of the MBZ/NSAID combination or mebendazole alone, as assessed by colonoscopy for patients prior to (but not delaying) prophylactic colectomy could yield a relatively rapid assessment of mechanism and efficacy in humans.

## MATERIALS AND METHODS

### Inhibition of CRC cell lines and xenografts with Mebendazole

Growth inhibition of MBZ on DLD-1, HCT-116, HT29 and SW480 colon cancer cell lines was measured by CCK-8 cell viability (Dijindo), as previously described [23]. HT29 and SW480 were each implanted subcutaneously into the flank of Athymic Nude mice, with growth factor-reduced matrigel (BD Sciences, San Jose, CA). After 5 days the mice were randomized into control and 50 mg/kg MBZ gavage treatment groups, n=5 mice per group. Mebendazole polymorph C tablets were crushed and mixed with 1:1 PBS/sesame oil and administered by oral gavage 5 days per week as previously described [23]. Tumor measurements were taken 2x per week with a digital calipers for 4 weeks of treatment.

### *Apc^Min/+^* mouse chemoprevention study

Heterozygous male C57BL/6J-Apc^Min^/J mice and wild-type female C57BL/6J mice (Jackson Laboratory, Bar Harbor, ME) were bred. The presence of the *Apc^Min/+^* mutation was confirmed in the tail snips of affected mice by Transnetyx, Inc (Cordova, TN). Affected pups were weaned at 21 days of age onto a mouse diet consisting of 45% kcal% fat (D12451, Research Diets), containing soybean oil and lard for fat. The mice were randomized into these groups: untreated control (n=12), 50 mg/kg MBZ gavage (n=7), 35 mg/kg MBZ in feed (n=12), 160 ppm sulindac (n=13), 50 mg/kg MBZ gavage + 160 ppm sulindac (n=5) or 35 mg/kg MBZ in feed + 160 ppm sulindac (n=19).

At 3 weeks of age, drinking water with 160 ppm (0.5 mg/day) Sulindac (Sigma) in 4 mM sodium phosphate buffered drinking water was supplied [18], as previously described as an effective dose [30, 31]. MBZ by gavage was 50 mg/kg for 5 days per week as previously described [23], starting at 5 weeks of age. MBZ was also administered by a custom feed of MBZ polymorph C in the high fat mouse diet, starting at 5 weeks of age. All animal experiments were performed under an approved protocol and in accordance with Johns Hopkins Animal Care and Use guidelines.

At 100 days of age, mice were euthanized and the intestines were removed, opened and the tumors counted with a dissecting microscope at 20x magnification in the colon and the proximal, middle, and distal thirds of the small intestines [41]. Individual polyps were measured with digital calipers and categorized for ≤ 1.0 mm, 1.0-2.0 mm, 2.1-3.0, and ≥ 3.0 mm in diameter. Tissue segments were swiss-rolled and fixed in 10% formalin [42] and scanned at 40X with a Hamamatsu Nanozoomer-XR digital slide scanner for histology and microadenomas.

### Immunohistochemical and immunofluorescent staining

Deparaffinized tissue sections were rehydrated and antigens were unmasked using citrate buffer and endogenous peroxidase and biotin were blocked. Immunohistochemical staining was on slides coated in 10% goat serum/1% BSA solution containing primary antibodies for Ki67 (1:500), MYC (Abcam, 1:500), COX2 (Cell Signaling, 1:500), BCL2 (Santa Cruz, 1:400), CD31 (Thermo Scientific, 1:50), and incubated overnight at 4°C. Super Sensitive Link-Label IHC biotin-conjugated/HRP secondary antibodies (Biogenex) and DAB chromogen substrate (Biogenex) were used for detection. Slides were counterstained with hematoxylin scanned at 20X using an Aperio AT2 slide scanner. For microvessel density (MVD), tumor sections were viewed under 20X magnification to identify microvessel “hotspots” [43] and the average MVD was calculated from 10 fields from each treatment group. Dual immunofluorescent visualization of vascular-endothelial growth factor receptor 2 inhibition was performed as previously described [23].

### Inflammatory cytokine immunoassay

Intestinal tissue lysates from each group were analyzed for pro-inflammatory cytokines and pro-angiogenic factors using a colorimetric Mouse ELISA strip (Signosis) containing TNF, IL6, VEGF, CCL2, IL1B, G-CSF, GM-CSF, and FGF2. Tissue protein concentration was determined using BCA protein assay and ELISA was performed using 10 μg protein/well as per manufacturer's instructions. Absorbance values were obtained on a VICTOR3 plate reader at 450 nm and graphed as fold difference versus the untreated control.

### Statistics

GraphPad Prism 5.0 software was used for all statistical analyses. Two-tailed Student's t-tests were used for comparisons between groups. Quantitative data are presented as mean and standard error of mean (SEM). Statistical analysis between treatment groups was determined by unpaired Student's t-test and P < 0.05 was considered statistically significant.
